# Harnessing unsupervised machine learning with [^18^F]FDG PET/CT to develop a composite model for predicting overall survival in cervical cancer patients undergoing concurrent chemoradiotherapy

**DOI:** 10.3389/fonc.2025.1486654

**Published:** 2025-05-02

**Authors:** Jinyu Shi, Lian Wang, Min Zhou, Shushan Ge, Bin Zhang, Jiangqin Han, Jihui Li, Shengming Deng

**Affiliations:** ^1^ Department of Nuclear Medicine, The First Affiliated Hospital of Soochow University, Suzhou, China; ^2^ Department of Oncology, Xuyi People’s Hospital, Huaian, China; ^3^ Department of Nuclear Medicine, Yancheng No.1 People’s Hospital, Affiliated Hospital of Medical School, Nanjing University, Yancheng, China; ^4^ Department of Nuclear Medicine, National Health Commission (NHC) Key Laboratory of Nuclear Technology Medical Transformation, Mianyang Central Hospital, Mianyang, Sichuan, China

**Keywords:** PET/CT, cervical cancer, unsupervised machine learning, concurrent chemoradiotherapy (CCRT), prognostic prediction

## Abstract

**Background and purpose:**

This study sought to develop an advanced composite model to enhance the prognostic accuracy for cervical cancer patients undergoing concurrent chemoradiotherapy (CCRT). The model integrated imaging features from [^18^F]FDG PET/CT scans with inflammatory markers using a novel unsupervised two-way clustering approach.

**Methods:**

In this retrospective study, 154 patients diagnosed with primary cervical cancer and treated with CCRT were evaluated using [^18^F]FDG PET/CT scans. A total of 1,702 radiomic features were extracted from the imaging data. These features underwent rigorous selection based on reproducibility and non-redundancy. The unsupervised two-way clustering method was then employed to simultaneously stratify patients and reduce the dimensionality of features, resulting in the generation of meta-features that were subsequently used to predict overall survival.

**Results:**

Kaplan-Meier survival analysis demonstrated that the two-way clustering method successfully stratified patients into distinct risk groups with significant survival differences (P<0.001), outperforming traditional K-means clustering. Predictive models constructed using meta-features derived from two-way clustering showed superior performance compared to those using principal component analysis (PCA), particularly when more than four features were included. The highest C-index values for the COX, COX_Lasso, and RSF models were observed with nine meta-features, yielding results of 0.691 ± 0.026, 0.634 ± 0.018, and 0.684 ± 0.020, respectively. In contrast, models based solely on clinical variables exhibited lower predictive performance, with C-index values of 0.645 ± 0.041, 0.567 ± 0.016, and 0.561 ± 0.033. The combination of clinical data, inflammatory markers, and radiomic features achieved the highest predictive accuracy, with a mean AUC of 0.88 ± 0.07.

**Conclusion:**

Integrating radiomic data with inflammatory markers using unsupervised two-way clustering offered a robust approach for predicting survival outcomes in cervical cancer patients. This methodology presented a promising avenue for personalized patient management, potentially leading to more informed treatment decisions and improved outcomes.

## 1 Introduction

Cervical cancer is the fourth most common cancer affecting women globally, representing a considerable public health issue, particularly in low- and middle-income countries (1, 2). Despite this, recent advances in medical treatment have positioned concurrent chemoradiotherapy (CCRT) as the standard of care for locally advanced cervical cancer, significantly enhancing survival rates when compared to radiotherapy alone (3, 4). However, if initial CCRT proves unsuccessful, the prolonged treatment course may hinder the timely implementation of alternative, potentially more effective therapies (5). Moreover, CCRT is associated with a range of adverse effects. For instance, extra-pelvic irradiation can affect bones that harbor a significant portion of the body’s actively proliferative bone marrow, raising the risk of myelosuppression (6, 7). Given these challenges, accurately predicting clinical outcomes is essential for tailoring personalized treatment strategies for cervical cancer patients at different risk levels and ensuring prompt intervention in high-risk cases.

[^18^F]Fluorodeoxyglucose ([^18^F]FDG) positron emission tomography (PET/CT) is widely employed in the diagnosis, clinical staging, and treatment monitoring of cervical cancer and other malignancies (8, 9). The standardized uptake value (SUV) derived from [^18^F]FDG-PET is particularly valuable, offering critical biological insights into tumor aggressiveness by reflecting parameters such as vascular function, cellularity, and glucose metabolism (10, 11). Additionally, numerous studies have demonstrated a strong correlation between inflammatory markers, such as neutrophil count (NC), C-reactive protein (CRP), and the neutrophil-to-lymphocyte ratio (NLR), and the prognosis of cervical cancer (12, 13). Thus, integrating data from these various modalities could potentially lead to a more accurate risk stratification, surpassing the precision currently achievable with single-modality assessments.

In recent years, the field of radiomics has experienced remarkable growth in the study of cervical cancer, particularly in predicting treatment responses, patient stratification, and prognosis using radiological imaging data (14). Radiomic features derived from PET images, in particular, have demonstrated strong potential in forecasting overall survival (OS) and disease-free survival in cervical cancer patients (15, 16). Additionally, several studies have underscored the utility of radiomics in predicting recurrence and metastasis in patients undergoing CCRT (17).

However, radiomics studies in cervical cancer face significant challenges, notably the high dimensionality that arises from small sample sizes coupled with extensive feature sets. This complexity necessitates the application of feature selection or dimensionality reduction techniques to improve predictive accuracy (18). While supervised feature selection methods are commonly employed, they often carry the risk of overfitting, and unsupervised techniques like principal component analysis (PCA) may not always deliver optimal results. To address these limitations, we introduced a novel unsupervised two-way clustering approach that not only reduced dimensionality but also generated meta-features by simultaneously sub-clustering both features and samples. This method effectively captured covariation among features and delineated distinct patterns across sample groups, providing a form of weak supervision that enhances the informativeness and utility of the feature representation (19).

Therefore, the primary objective of this study was to develop a composite model for predicting the prognosis of cervical cancer patients undergoing CCRT. This model integrated PET/CT imaging features with inflammatory markers through the application of an innovative, unsupervised two-way clustering method.

## 2 Materials and methods

### 2.1 Patients

**Figure 1:**
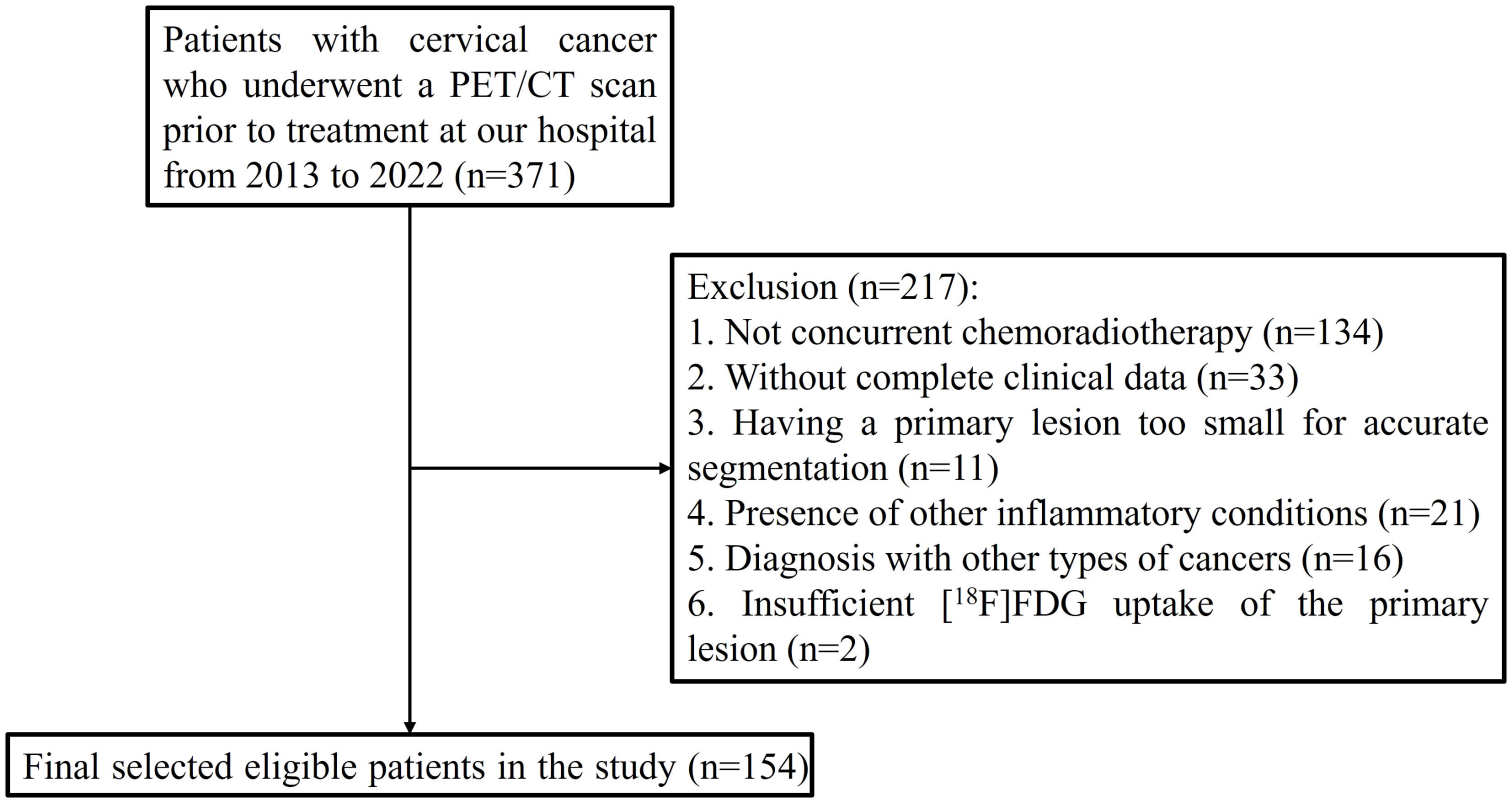
Flowchart of selection of cervical cancer patients.

This retrospective study received approval from the institutional review board of the First Affiliated Hospital of Soochow University, with informed consent being waived due to the study’s retrospective nature. The research was conducted in accordance with the Declaration of Helsinki, and the trial registration number is (2024) Ethical Research Approval No. 305. From June 2013 to June 2022, a total of 154 participants with histologically confirmed primary cervical cancer were included in the study, all of whom underwent [^18^F]FDG PET/CT scans for staging purposes. The inclusion criteria were (1) age 18 years or older, (2) no prior treatment before the initial [^18^F]FDG PET/CT scan, (3) histological confirmation of the cancer type and grade, obtained via biopsy (typically for larger lesions) or surgical specimens (commonly for smaller lesions), and (4) treatment with CCRT. The exclusion criteria included (1) the absence of CCRT, (2) incomplete clinical data, (3) a primary lesion too small for accurate segmentation, (4) the presence of other inflammatory conditions, (5) a diagnosis of other types of cancers, and (6) insufficient [^18^F]FDG uptake of the primary lesion, making accurate lesion delineation unfeasible (**Figure 1**).

### 2.2 Inflammatory marker assessment

In this study, a panel of inflammatory markers, including white blood cell (WBC) count, NC, lymphocyte count (LC), platelet count (PLT), and the NLR (defined as NC divided by LC), was systematically assessed as part of the patients’ baseline hematological profile. These indicators were selected to examine their potential correlations with clinical disease features and patient prognoses. All parameters were measured using standardized laboratory protocols, and their prognostic significance in relation to survival outcomes was subsequently analyzed.

### 2.3 [^18^F]FDG PET/CT imaging

In this study, patients received an injection of [^18^F]FDG at a dose ranging from 4.07 to 5.55 MBq/kg after fasting for a minimum of 6 hours to maintain blood glucose levels below 11.1 mmol/L. Approximately 40 to 60 minutes post-injection, imaging was conducted using an integrated PET/CT scanner (Discovery STE, General Electric Medical Systems, Milwaukee, WI, USA), which scanned from the base of the skull to the midthigh. The imaging parameters included a 70-cm transaxial field of view, a pitch of 1.75, a rotation time of 0.8 seconds, and a slice thickness of 3.75 mm. Low-dose CT images were acquired at 140 kV and 120 mA, serving dual purposes: attenuation correction and providing an anatomical reference. This was immediately followed by PET scans, conducted over 2 to 3 minutes per bed position. The images were then reconstructed using the ordered subset expectation maximization (OSEM) algorithm to ensure the generation of high-quality diagnostic images.

### 2.4 Feature extraction

PET images were analyzed using 3D Slicer software (version 5.2.2, available at http://www.slicer.org). The images were reviewed in axial, coronal, and sagittal planes, including both standalone CT and combined PET/CT images, by two experienced nuclear medicine physicians to accurately identify the primary cervical cancer lesions. These specialists manually delineated the lesions to ensure precision. Key semi-quantitative metrics from the PET/CT scans, including the maximum standardized uptake value (SUV_max_), mean standardized uptake value (SUV_mean_), and peak standardized uptake value (SUV_peak_, automatically calculated as the highest average SUV within a 1.0 cm³ spherical volume of the tumor), were meticulously measured.

When the PET and CT images were well-aligned, the volume of interest (VOI) delineated on the PET images was mirrored onto the CT images to define the corresponding CT VOI. A total of 1,702 image features were extracted, comprising 107 general features (**Table 1**) and 744 filtered features from both CT and PET images, creating a comprehensive dataset for subsequent analysis. All PET/CT scans were independently reviewed and manually segmented by two seasoned nuclear medicine physicians (L.J. and X.X.), who possessed 10 and 12 years of diagnostic experience with PET/CT, respectively. Each physician delineated the lesions for all patients in a blinded fashion to ensure objectivity. To minimize variability arising from differences in segmentation approaches (anatomical versus metabolic), only radiomic features demonstrating high inter-observer consistency (intraclass correlation coefficient [ICC] > 0.75) were retained for further analysis (Section 2.5 for additional details).

**Table 1:** The list of extracted radiomic features.

Shape (14)	First order (18)	GLCM (24)	GLDM (14)	GLRLM (16)	GLSZM (16)	NGTDM (5)
Elongation Flatness Least axis Major axis M2DDC M2DDR M2DDS M3DD Mesh volume Minor axis Sphericity Surface Area SVR Voxel volume	10Percentile 90Percentile Energy Entropy Interquartile range Kurtosis Maximum MAD Mean Median Minimum Range RMAD RMS Skewness Total energy Uniformity Variance	Autocorrelation Cluster prominence Cluster shade Cluster tendency Contrast Correlation Difference average Difference entropy Difference variance Id Idm Idmn Idn Imc1 Imc2 Inverse variance Joint average Joint energy Joint entropy MCC Max Probability Sum average Sum entropy Sum squares	Dependence entropy DNU DNUN Dependence variance GLNU GLV HGLE LDE LDHGLE LDLGLE LGLE SDE SDHGLE SDLGLE	GLNU GLNUN GLV HGLRE LRE LRHGLE LRLGLE LGLRE Run entropy RLNU RLNUN Run percentage Run Variance SRE SRHGLE SRLGLE	GLNU GLNUN GLV HGZE LAE LAHGLE LALGLE LGLZE SZNU SZNUN SAE SAHGLE SALGLE Zone entropy Zone percentage Zone variance	Busyness Coarseness Complexity Contrast Strength

A full list of abbreviations used in this table is provided in **Supplementary Table S1**.

### 2.5 Selection of radiomic features

To assess the consistency between observers, an interclass correlation coefficient (ICC) analysis was conducted on 20 randomly selected cases, independently delineated by two researchers, L.J. and X.X., who had 10 and 12 years of experience in PET/CT diagnostics, respectively. Radiomic features with an ICC greater than 0.75 were deemed reproducible. Additionally, a pairwise Pearson correlation matrix (PCM) was employed to identify pairs of highly correlated features (|r| ≥ 0.76 for PET and |r| ≥ 0.4 for CT) within inner clusters, ensuring feature non-redundancy. The results were visually represented in a heatmap to facilitate comparative analysis. This rigorous selection process identified 64 imaging features as significant, comprising 12 features from CT images and 52 from PET images (**Table 2**).

**Table 2:** The list of selected features.

Category	Selected features
PET features	Elongation Flatness Least axis Sphericity 10Percentile 90Percentile Kurtosis Minimum Cluster shade Id DNUN LDLGLE GLCM_Contrast Skewness Correlation MCC wavelet-LLH_glcm_Cluster shade wavelet-LLH_firstorder_Kurtosis wavelet-LHL_firstorder_Median wavelet-LHL_firstorder_Skewness wavelet-LHL_glcm_Cluster shade wavelet-LHL_gldm_Dependence variance wavelet-LHH_firstorder_Kurtosis wavelet-LHH_firstorder_Mean wavelet-LHH_glcm_Cluster tendency wavelet-LHH_glcm_Correlation wavelet-LHH_glcm_Imc2 wavelet-LHH_gldm_LGLE wavelet-LHH_glrlm_GLV wavelet-HLL_firstorder_Kurtosis wavelet-HLL_firstorder_Range wavelet-HLL_glcm_Cluster shade wavelet-HLL_glcm_Correlation wavelet-HLL_glrlm_HGLRE wavelet-HLH_firstorder_Median wavelet-HLH_firstorder_Skewness wavelet-HLH_glcm_Correlation wavelet-HLH_gldm_SDE wavelet-HLH_gldm_LGLE wavelet-HLH_glrlm_RunVariance wavelet-HHL_firstorder_Mean wavelet-HHL_firstorder_Maximum wavelet-HHL_glcm_Correlation wavelet-HHL_gldm_LDLGLE wavelet-HHL_glszm_GLNU wavelet-HHH_firstorder_Kurtosis wavelet-HHH_firstorder_Range wavelet-HHH_firstorder_Skewness wavelet-HHH_glcm_Cluster shade wavelet-HHH_gldm_GLNU wavelet-HHH_glrlm_LRE wavelet-LLL_gldm_DNUN
CT features	Elongation Least axis 10Percentile Kurtosis wavelet-LHL_firstorder_Mean wavelet-LHH_glcm_Difference average wavelet-LHH_firstorder_Median wavelet-HLL_glcm_Idn wavelet-HLH_glcm_MCC wavelet-HHL_glcm_InverseVariance wavelet-HHH_firstorder_Mean wavelet-LLL_firstorder_90Percentile

For detailed definitions of abbreviations, please refer to **Supplementary Table S2**.

### 2.6 Unsupervised two-way clustering analysis

**Figure 2:**
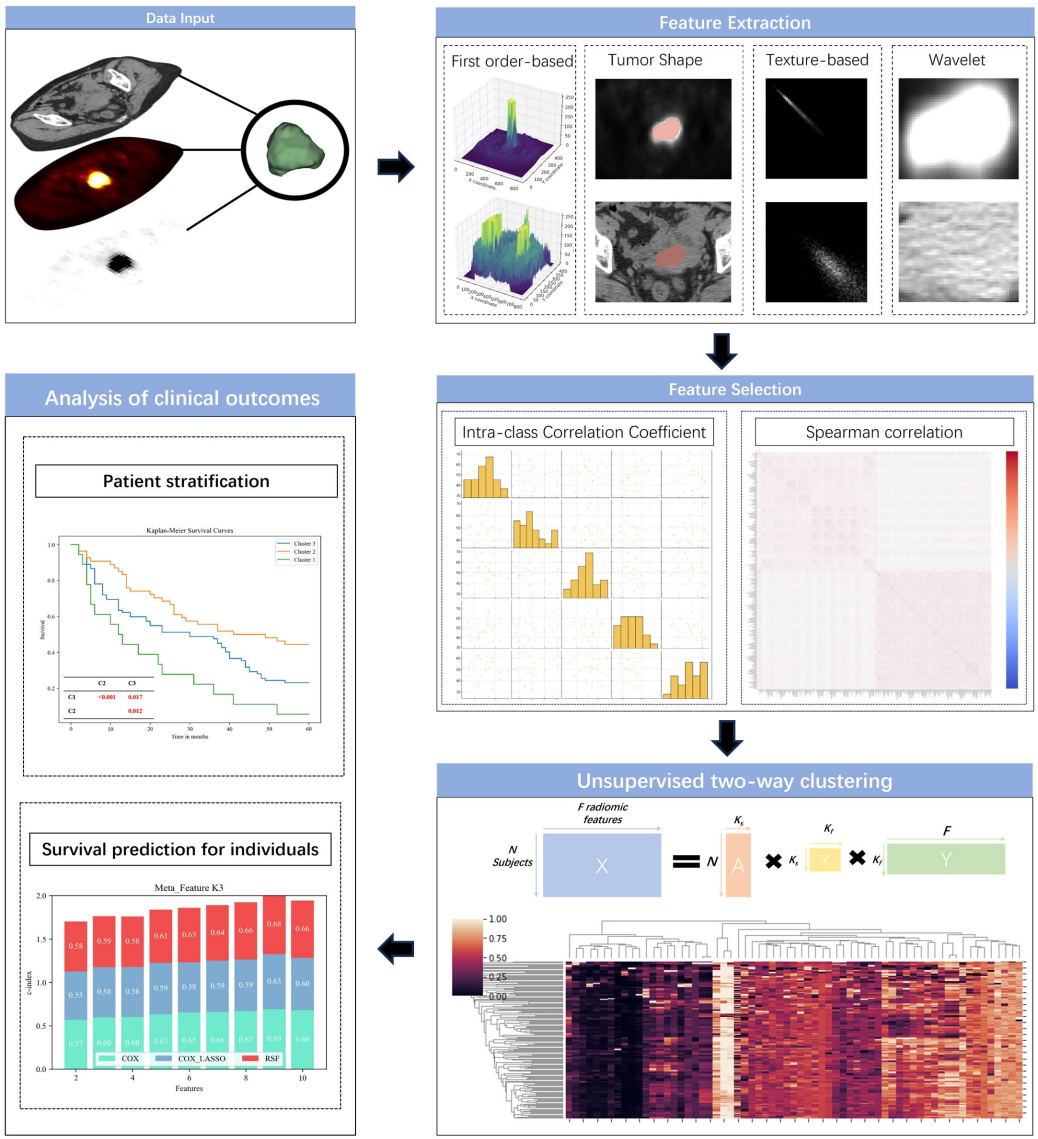
Outline of the predictive modeling workflow for unsupervised learning of radiomic signatures.

To simultaneously cluster patients and radiomic features into sub-clusters, we utilized an unsupervised two-way clustering approach, leveraging a matrix tri-factorization technique. Given the feature matrix X∈R^N×F^, where N represents the number of patients and F is the number of radiomic features, matrix tri-factorization decomposes X into three low-rank matrices A, S, and Y. This decomposition minimizes the approximation error. $\frac{\min}{A,S,Y}||X-ASY|{|}_{F}^{2}$ ,s.t. A≥0, S≥0, Y≥0, A^T^A=I, YY^T^=I, where I is an identify matrix. As illustrated in **Figure 2**, the low-rank matrix A∈ $R_{+}^{N\times K_{S}}$ encodes the membership of K_s_ sub-clusters of patients, matrix Y∈ $R_{+}^{K_{f}\times F}$ encodes the membership of K_f_ subclusters of features, matrix S∈ $R_{+}^{K_{S}\times K_{f}}$ encodes scales of different data points as well as interactions between A and Y. The parameters K_s_ and K_f_, which define the number of sub-clusters for patients and features, were predetermined before the clustering process. The optimization challenge was tackled using an alternating optimization strategy. Upon obtaining the decomposition results, the low-dimensional meta-features M∈ $R_{+}^{N\times K_{f}}$ were calculated as M = AS. These meta-features were subsequently employed to construct prediction models aimed at forecasting clinical outcomes (**Figure 2**).

### 2.7 Patient stratification methods and evaluation

Based on the patient clustering results and the meta-features generated through the unsupervised two-way clustering approach, we performed patient stratification and predicted OS. To assess the effectiveness of the two-way clustering method in stratifying patients, we used Kaplan-Meier estimation to calculate survival functions for each patient group, with differences between groups analyzed using the Log-rank test. We explored two configurations for patient sub-clustering: setting the number of sub-clusters (K_s_) to 2 and 3. We hypothesized that two sub-clusters might effectively distinguish patients into low- and high-mortality risk groups, while three sub-clusters could further differentiate them into low-, medium-, and high-risk categories. The number of meta-features was fixed at 9, a value determined through a cross-validation approach that demonstrated optimal performance in survival prediction.

### 2.8 Construction of survival prediction model

To predict each patient’s risk of mortality, the meta-features extracted from the unsupervised two-way clustering were used to build prediction models employing three distinct survival modeling techniques: Cox proportional hazard regression (Cox regression), Cox regression with Lasso (Cox_Lasso), and random survival forests (RSF). The Cox regression, a widely used method in survival analysis, serves as a standard semi-parametric model, while Cox_Lasso, also a semi-parametric model, incorporates feature selection during model training to improve predictive accuracy. In contrast, the RSF method is a fully non-parametric approach that not only predicts survival outcomes but also identifies the most informative features. Before model development, the complete cohort of 154 patients was randomly partitioned into a training set (n = 108) and a validation set (n = 46), adhering to an approximate 7:3 ratio. The training set served to construct the survival prediction models and optimize their hyperparameters, while the validation set was exclusively utilized to assess model generalizability. This data-splitting approach was uniformly applied across all modeling strategies to ensure equitable comparisons and a rigorous evaluation of predictive performance.

We explored various parameter settings for K_s_ and K_f_ within the two-way clustering method to assess their impact on the prediction performance. This thorough evaluation aimed to optimize the model’s predictive capabilities, ensuring that the most effective combination of parameters was utilized for accurate mortality risk prediction.

Specifically, the number of patient sub-clusters (K_s_) was fixed at 3, while the number of feature sub-clusters (K_f_) was varied from 2 to 10 in increments of 1, which was deemed an appropriate range given the study’s sample size of 154 patients. All prediction models were trained and evaluated using a consistent 3-fold cross-validation framework. The concordance index (c-index) was used as the metric to assess the predictive performance of these models. To ensure robustness, this cross-validation procedure was repeated 100 times, with the results reported as the mean and standard deviation of the c-index.

The prediction models were developed using the CoxPHFitter and RandomSurvivalForest modules in Python. For the Cox_Lasso method, the sparsity parameter was automatically determined through a nested 3-fold cross-validation process to optimize feature selection and model performance. In the RSF model, 500 decision trees were employed, with a minimum leaf size set to 5, to enhance the model’s precision and stability in survival prediction.

### 2.9 Method comparison and validation

To thoroughly assess the effectiveness of our proposed method, we benchmarked it against several alternative techniques, adding a layer of comparative analysis to validate our approach. For patient stratification, we contrasted our method with K-means clustering, applying the same set of 64 radiomic features under identical conditions. This comparison aimed to highlight the superiority of our technique in accurately categorizing patients into distinct risk groups.

In terms of feature dimensionality reduction, we pitted our method against PCA. PCA was employed to generate low-dimensional feature representations, which were then used to construct survival prediction models. The predictive accuracy of the PCA-derived features was rigorously tested using the same cross-validation framework we applied to our method.

To ensure that our models were constructed with optimal parameters, we utilized a nested 5-fold cross-validation approach. This process meticulously optimized the parameter combinations (K_s_ = 3; K_f_ ∈ [2, 10]) based on training data, maximizing the model’s performance. Similarly, for PCA-based feature extraction, the optimal number of features was determined within the range of 2 to 10, following the same rigorous method.

In addition, we developed prediction models using traditional clinical variables, such as age, body mass index (BMI), T stage, inflammatory markers, and semi-quantitative PET/CT parameters, to predict individual mortality risk. These models underwent evaluation through a 3-fold cross-validation process, repeated 100 times to guarantee robustness and reliability. The results were reported as the mean prediction performance across these iterations, providing a comprehensive and rigorous comparison that underscored the advantages of our method over conventional techniques. This multi-layered benchmarking not only reinforced the validity of our approach but also demonstrated its potential as a superior tool for patient stratification and survival prediction.

## 3 Results

### 3.1 Baseline demographic information

The baseline characteristics of the study population are detailed in **Table 3**, offering a comprehensive overview of the 154 patients recruited for this study. The average age of the participants was 54.88 ± 12.99 years, with a mean BMI of 22.42 ± 1.41 kg/m². The mean SUV_max_ recorded was 15.01 ± 6.67, reflecting the metabolic activity of the tumors. The mean OS for the cohort was 34.72 ± 22.38 months, with survival times ranging from 2 to 60 months.

**Table 3:** Characteristics of patients in this study.

Age (years)			54.88 ± 12.99
BMI (kg/m^2^)			22.42 ± 1.41
SUV_max_			15.01 ± 6.67
Lymph node metastasis	(positive) (negative)		126 (81.82%) 28 (18.18%)
SCCA	(positive) (negative)		107 (69.48%) 47 (30.52%)
CEA	(positive) (negative)		93 (60.39%) 61 (39.61%)
CA-19.9	(positive) (negative)		57 (37.01%) 97 (62.99%)
CA-125	(positive) (negative)		78 (50.65%) 76 (49.35%)
WBC (1×10^9^/L)			7.20 ± 2.74
NC (1×10^9^/L)			5.16 ± 2.47
LC (1×10^9^/L)			1.53 ± 0.53
PLT (1×10^9^/L)			263.44 ± 138.87
NLR			3.85 ± 2.55
T stage	(T2)		59 (38.31%)
	(T3)		60 (38.96%)
	(T4)		35 (22.73%)
FIGO	(II)	(IIA)	28(18.18%)
		(IIB)	31(20.13%)
	(III)	(IIIA)	38(24.68%)
		(IIIB)	22(14.29%)
	(IV)	(IVA)	35(22.73%)
Histology	(adenocarcinoma)		8 (5.19%)
	(squamous cell carcinoma)		146 (94.81%)

BMI, Body Mass Index; SUV_max_, maximum standardized uptake value; WBC, White blood cells; NC, Neutrophil count; LC, Lymphocyte count; PLT, Platelets; NLR, Neutrophil-to-lymphocyte ratio; SCCA, Squamous cell carcinoma antigen; CEA, Carcinoembryonic antigen; CA-19.9, Carbohydrate antigen 19.9; CA125, Carbohydrate antigen 125.

Lymph node metastasis was present in a significant majority of patients, with 126 individuals (81.82%) testing positive, while 28 patients (18.18%) were negative. Regarding the squamous cell carcinoma antigen (SCCA), 107 patients (69.48%) showed positive results, compared to 47 patients (30.52%) who were negative. Similarly, carcinoembryonic antigen (CEA) was positive in 93 patients (60.39%), leaving 61 patients (39.61%) negative.

The hematological profile of the patients revealed a mean WBC count of 7.20 ± 2.74×10^9^/L, a mean NC of 5.16 ± 2.47×10^9^/L, and a mean LC of 1.53 ± 0.53×10^9^/L. Tumor staging showed that 59 patients (38.31%) were classified as T2, 60 patients (38.96%) as T3, and 35 patients (22.73%) as T4. The histological analysis identified that eight patients (5.19%) had adenocarcinoma, while the overwhelming majority, 146 patients (94.81%), were diagnosed with squamous cell carcinoma. This dataset provided a detailed snapshot of the study cohort, encompassing key demographic, clinical, and pathological features that were pivotal in the subsequent analysis and model development.

### 3.2 Patient stratification

**Figure 3:**
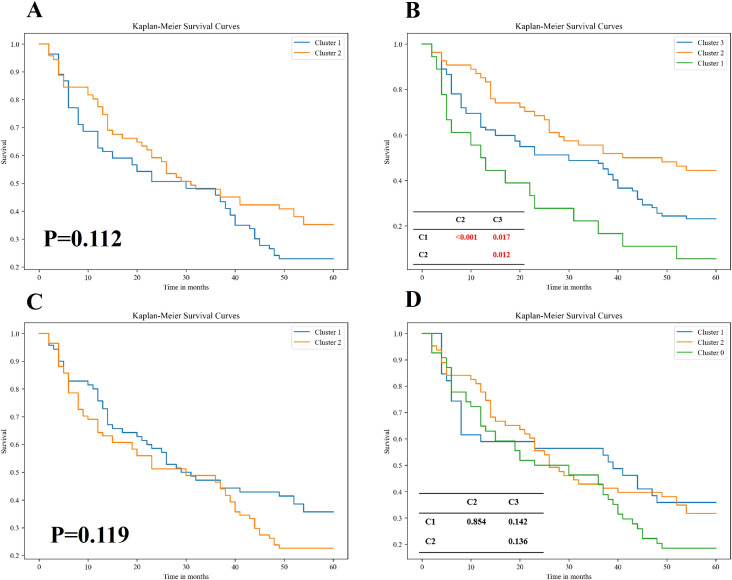
Kaplan–Meier survival analysis of patient subgroups stratified by two distinct unsupervised clustering algorithms. **(A, B)** Survival curves for patients grouped using an unsupervised bidirectional clustering approach, with two **(A)** and three **(B)** clusters, respectively. **(C**, **D**) Survival curves for patients stratified by K-means clustering, also set to two **(C)** and three **(D)** clusters, respectively. Statistical comparisons of survival distributions between subgroups were performed using the log-rank test. In panels **(B, D)**, pairwise p-values are provided in the corresponding tables to illustrate intergroup differences in overall survival among Cluster 1, Cluster 2, and Cluster 3. Notably, the bidirectional clustering approach **(B)** demonstrated statistically significant separation among clusters, whereas K-means clustering **(D)** did not yield significant intergroup differences.

**Figure 3** presents the Kaplan-Meier survival plots for patients stratified by the unsupervised two-way clustering method into two groups (**Figure 3A**) and three groups (**Figure 3B**). When patients were divided into two groups, the Log-rank test revealed no statistically significant difference in survival rates between the two groups (P=0.112), although a visual inspection of the Kaplan-Meier plots suggested a noticeable difference. However, when stratified into three groups, the survival differences became more pronounced. The low-risk mortality group (orange curve) showed a statistically significant survival advantage over the high-risk group (green curve) (P< 0.001). Additionally, both the low-risk group and the high-risk group demonstrated statistically significant differences in mortality compared to the medium-risk group (blue curve) (P=0.012; P=0.017; **Figure 3B**). These findings suggested that the two-way clustering method effectively distinguished patients with varying clinical outcomes, providing meaningful stratification based on survival.

In contrast, **Figures 3C, D** display the results of patient stratification achieved by applying K-means clustering to the original radiological features. Similar to the two-way clustering method, dividing patients into two groups resulted in no significant differences in survival rates (P=0.119). However, unlike the two-way clustering, dividing patients into three groups using K-means did not yield statistically significant differences in survival among the groups. This indicated that traditional K-means clustering was less effective than the two-way clustering method in stratifying patients for predicting survival outcomes, highlighting the superior performance of the two-way clustering approach in this context.

### 3.3 Representative cases

**Figure 4:**
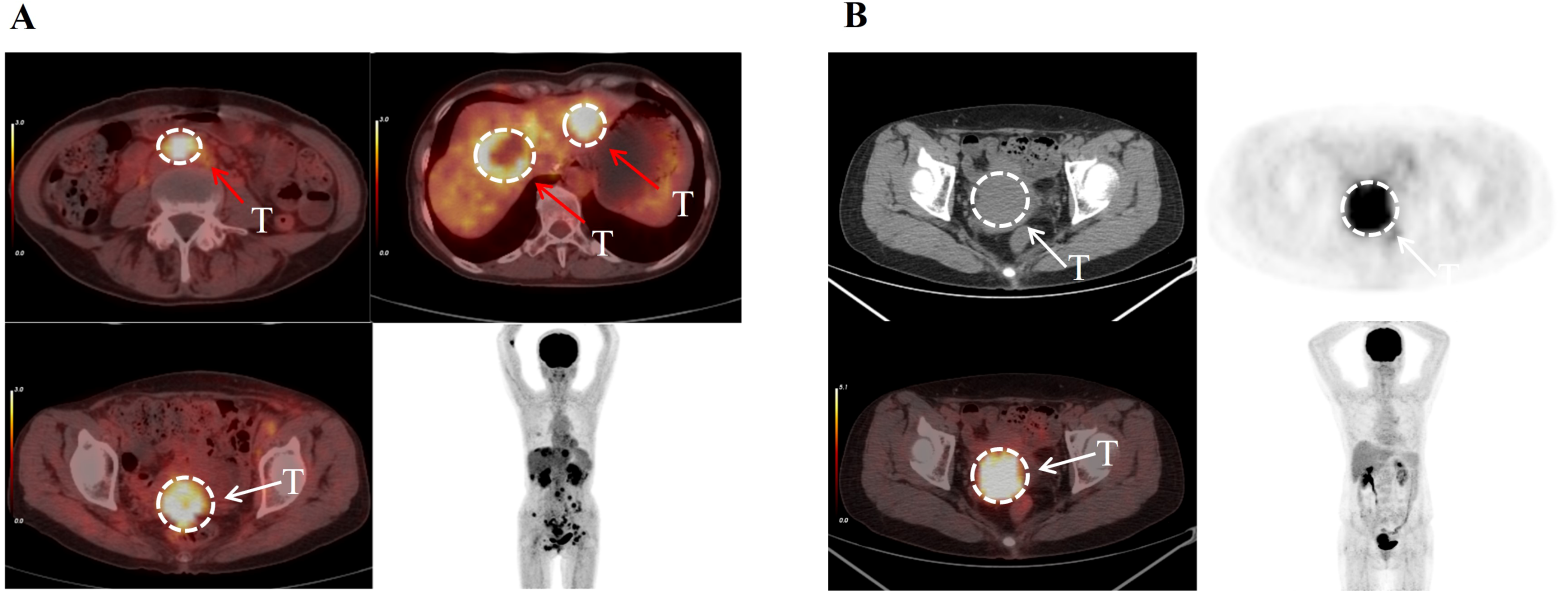
The patient depicted in **(A)**, aged 58, was diagnosed with cervical cancer characterized by multiple metastases. Her disease was classified as Stage IV according to the FIGO system. Despite this, unsupervised two-way clustering analysis of her imaging features categorized her into the low-risk group, and she achieved an overall survival of 37 months. The patient shown in **(B)**, aged 56, was diagnosed with primary cervical cancer without metastases. Her condition was staged as II according to the FIGO classification. Despite this staging, she was categorized as high-risk, with an actual overall survival of only 4 months. This suggests that unsupervised two-way clustering analysis can identify risk groups that may not be apparent through traditional clinical assessments. The white arrows indicate the primary cervical tumors, while the red arrows indicate distant metastatic lesions.

The patient described in **Figure 4A** was diagnosed with cervical cancer accompanied by extensive systemic metastasis. However, after receiving CCRT treatment, the patient’s overall survival (OS) reached an impressive 37 months. This patient was classified as being in the low-risk group based on unsupervised two-way cluster analysis of radiomic features. In contrast, the patient shown in **Figure 4B** was diagnosed with cervical cancer without systemic metastasis, but was classified as being in the high-risk group with an OS of only 4 months. These contrasting cases highlight the ability of our unsupervised two-way clustering hierarchical model to accurately identify patients with advanced disease and high prognostic risk, thereby demonstrating the model’s potential utility in guiding personalized treatment strategies.

### 3.4 Prediction of OS

**Figure 5:**
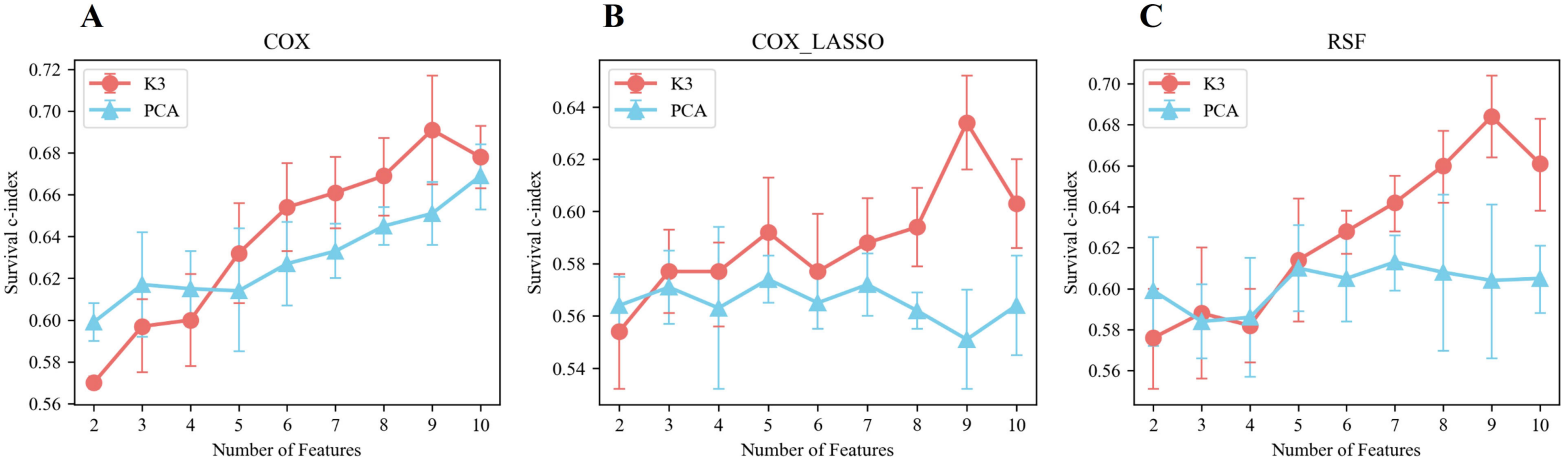
Performance of prediction models built based on meta-features extracted by unsupervised bidirectional clustering (K3) and PCA in terms of survival using different prediction models. **(A)** Cox regression model. **(B)** LASSO-penalized Cox regression model. **(C)** Random survival forest (RSF) model.

The prediction performances of the various models are illustrated in **Figure 5**, revealing key insights into the effectiveness of different feature extraction methods. Notably, the prediction model constructed using meta-features derived from the unsupervised two-way clustering method consistently outperformed the model that utilized PCA for dimensionality reduction, particularly when the number of features exceeded four. A significant finding was that when nine meta-features were extracted through the two-way clustering method, the C-index values for the three models, COX, COX_Lasso, and RSF, reached their optimal levels, with values of 0.691 ± 0.026, 0.634 ± 0.018, and 0.684 ± 0.020, respectively.

When examining scenarios where the number of features exceeded four, the survival C-indexes for the Cox, Cox_Lasso, and RSF models based on meta-features from two-way clustering were 0.794 ± 0.025, 0.716 ± 0.027, and 0.761 ± 0.015 in the training set, and 0.664 ± 0.020, 0.598 ± 0.022, and 0.648 ± 0.025 in the validation set, respectively. In contrast, using PCA-derived features, the survival C-indexes in the training set for the same models were 0.702 ± 0.031, 0.679 ± 0.022, and 0.698 ± 0.015, while in the validation set, they were 0.640 ± 0.019, 0.565 ± 0.008, and 0.608 ± 0.004, respectively.

Overall, these results strongly indicated that the best-performing survival prediction models were those built upon the meta-features extracted using the two-way clustering method, demonstrating its superior ability to enhance predictive accuracy over conventional PCA-based approaches.

**Figure 6:**
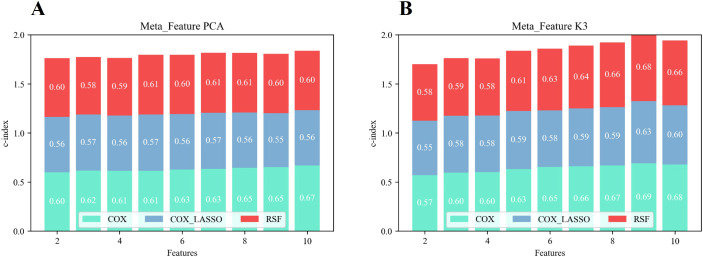
Overall predictive performance in terms of survival of the prediction model based on PCA features **(A)** and two-way clustering features **(B)**.

**Figure 6** illustrates the overall predictive performance of models using different meta-features derived from unsupervised two-way clustering and PCA-based dimensionality reduction. The calculation was based on the sum of the C-index values from the Cox, Cox_Lasso, and RSF prediction models across varying numbers of features. The results demonstrated that when the number of meta-features was five or more, the predictive performance of the extracted meta-features consistently exceeded that of the PCA-derived features. This suggested that the meta-features generated through two-way clustering provided more valuable information for predicting survival outcomes. Notably, the overall C-index reached its maximum value of 2.009 when the number of meta-features was limited to nine, surpassing the performance of the PCA-derived features, which achieved a C-index of 1.806 (p< 0.05). This result highlighted the nine-feature meta-set as the optimal configuration for survival prediction.

In comparison, prediction models built solely on clinical variables performed significantly worse. In the training cohort, the C-index values of the Cox, Cox_Lasso, and RSF models were 0.659 ± 0.038, 0.607 ± 0.019, and 0.613 ± 0.031, respectively. In the validation cohort, the corresponding C-index values were 0.645 ± 0.041, 0.567 ± 0.016, and 0.561 ± 0.033, respectively (p< 0.05). These values were considerably lower than those obtained from models based on radiological features, highlighting the superior predictive power of the radiomic features over traditional clinical variables. This underscored the importance of integrating advanced imaging-derived features into survival prediction models to achieve more accurate and informative outcomes.

### 3.5 Receiver operating characteristic curves and feature combination analysis

**Figure 7:**
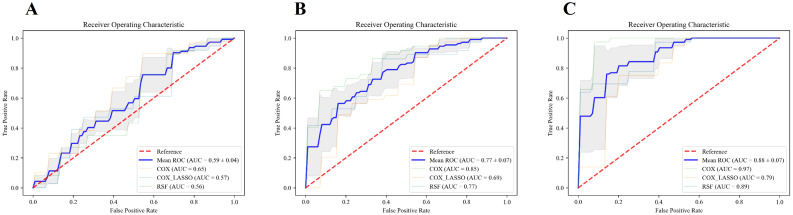
ROC curves for different feature sets and models. **(A–C)** illustrate the ROC curves of the models constructed using traditional clinical features alone, the combination of traditional clinical features and inflammatory indicators, and the combination of clinical features, inflammatory indicators, and radiomic features, respectively. The blue line represents the average AUC value of the three models, COX, COX_Lasso, and RSF.

**Figure 7** presents the ROC curves for models built using various combinations of features, highlighting the impact of integrating different data types on predictive performance. In Panel A, the model relying solely on traditional clinical data achieved a mean area under the curve (AUC) of 0.59 ± 0.04 in the validation set, reflecting modest predictive accuracy, and the corresponding training set AUC was 0.64 ± 0.05. As illustrated in Panel B, incorporating inflammatory markers into the clinical dataset led to a marked improvement in model performance, with the mean AUC increasing to 0.77 ± 0.07 on the validation set and 0.83 ± 0.06 on the training set. The most pronounced enhancement was observed in Panel C, where the integration of clinical variables, inflammatory markers, and radiomic features produced the highest predictive accuracy, achieving a mean AUC of 0.88 ± 0.07 in the test set and 0.93 ± 0.03 in the training set. These results clearly demonstrated the cumulative benefits of integrating multiple feature sets. Adding inflammatory markers to clinical data substantially boosted the model’s accuracy, and including radiomic features further elevated the predictive power. The combination of all three types of features, clinical data, inflammatory markers, and radiomic features, produced the most precise survival predictions, underscoring the value of a comprehensive, multi-modal approach in prognostic modeling.

## 4 Discussion

Radiomics analysis has emerged as a powerful tool for quantitatively exploring the relationship between imaging data and clinical outcomes, offering a nuanced approach to understanding disease characteristics (20–22). The extraction of high-dimensional features from imaging data presents a unique challenge, particularly in radiomics studies with smaller sample sizes, where feature selection and dimensionality reduction become crucial for ensuring the reliability and robustness of the results (23–25). In our present study, we employed an innovative, unsupervised two-way clustering technique to predict OS in cervical cancer patients undergoing CCRT. This approach not only facilitated simultaneous patient stratification but also effectively managed feature dimensionality reduction. We hypothesized that by integrating patient stratification with feature reduction, we could enhance the analytical power of the study.

The experimental results supported this hypothesis, demonstrating that our method was highly competitive in both patient stratification and survival prediction when compared to traditional techniques. Notably, the prediction models incorporating radiological features significantly outperformed those relying solely on clinical indicators, underscoring the superior predictive value of imaging-derived data in determining patient outcomes. This finding highlighted the potential of radiomics as a critical component in developing more precise and personalized treatment strategies for cervical cancer patients.

In recent years, there has been a growing interest in integrating clinical data with imaging features to improve the prediction of lymph node metastasis, treatment response, and overall prognosis in various cancers, including cervical cancer (26, 27). For instance, Fang et al. have conducted a retrospective analysis of pre-treatment MRI images from 120 cervical cancer patients undergoing CCRT to predict tumor response, achieving AUCs of 0.820 and 0.798 in the training and internal validation sets, respectively (28). Similarly, Xu et al. have developed a CT-based hybrid radiomics nomogram for predicting OS in cervical cancer patients receiving CCRT, reporting AUCs of 0.871 in the training set and 0.730 in the internal validation set (29). Zhang et al. have adopted a different approach by employing a LASSO-Cox model to predict PFS based on MRI imaging characteristics and clinical data collected before CCRT treatment, with C-indexes of 0.792 and 0.809 for the training and internal validation sets, respectively (17). These studies, much like our own, underscore the significant potential of radiomics in enhancing prediction models through the integration of detailed imaging features. Notably, the integrated model developed in our study, encompassing clinical data, inflammatory markers, and radiomic features, demonstrated outstanding predictive performance, achieving AUCs of 0.93 ± 0.03 in the training set and 0.88 ± 0.07 in the validation set. These results surpassed those reported in comparable studies, underscoring the robustness and clinical utility of the multimodal approach.

However, it is important to note that the prediction models in these cited studies primarily rely on supervised machine learning techniques, where feature selection is a critical step in improving model performance. This selection process typically focuses on features that demonstrate high repeatability and strong discriminatory power (18, 30). While these features are indeed valuable for prediction, their selection can introduce a risk of overfitting, especially in studies with limited sample sizes. On the other hand, unsupervised dimensionality reduction techniques, such as PCA, are adept at identifying underlying relationships among raw features but fall short when it comes to prognostic tasks. This limitation arises because unsupervised methods do not take clinically relevant endpoint data into account during feature extraction, making them less effective for tasks that require a direct link between features and clinical outcomes.

Our study addressed this gap by employing an unsupervised two-way clustering approach. This approach simultaneously managed feature selection and patient stratification, thereby reducing the risk of overfitting while enhancing the model’s prognostic accuracy. This method allowed us to uncover clinically meaningful patterns in the data that might be overlooked by more traditional approaches, providing a robust framework for predicting patient outcomes in cervical cancer.

In this study, we introduced an innovative, unsupervised two-way clustering method that simultaneously performed patient stratification and feature dimensionality reduction (meta-feature extraction). This approach is grounded in the premise that the two processes mutually reinforce each other, leading to enhanced analytical outcomes. The patient stratification process in our method provided a form of weak supervision, which facilitated the extraction of features that were particularly informative for predicting clinical endpoints. These refined features, in turn, contributed to more precise and effective patient stratification. The survival analysis results for cervical cancer patients clearly demonstrated the superiority of our method over traditional approaches, where patient stratification and meta-feature extraction were typically carried out independently. Our method, when compared to the conventional K-means clustering technique, revealed more distinct survival differences across various patient groups, underscoring the significant advantages of integrating meta-feature extraction with patient stratification. Moreover, when it came to predictive performance, the meta-features derived through two-way clustering consistently outperformed those obtained via the PCA method across different feature dimensions and prediction model configurations. This finding highlighted the value of the weak supervision inherent in patient stratification, which enhanced the overall predictive accuracy of the model. By leveraging this integrated approach, our method not only improved the reliability of the predictions but also offered a more comprehensive understanding of the underlying patterns within the data, ultimately leading to better-informed clinical decision-making.

The inclusion of inflammatory markers such as NC, CRP, and NLR in our predictive model was consistent with recent research that highlights their prognostic significance in cervical cancer (12, 31–33). These markers reflect systemic inflammatory responses, which have been closely associated with tumor progression and poor outcomes across various malignancies. By combining these biomarkers with radiomic signatures, our study not only reinforced the importance of inflammation in cancer prognosis but also underscored its critical role in shaping tumor behavior, particularly in the context of CCRT.

However, several limitations warrant consideration. The retrospective design of this study, along with its reliance on data from a single institution, might limit the broader applicability of our findings. Furthermore, the computational complexity inherent in the two-way clustering analysis could present practical challenges for routine implementation in clinical settings. To address these limitations, future research should focus on validating these results through prospective multicenter trials and investigating the feasibility of incorporating this advanced analytical method into standard clinical workflows. Such efforts will be crucial in determining the true potential of this approach in improving patient outcomes on a larger scale.

## 5 Conclusion

In conclusion, our study demonstrated the significant potential of leveraging unsupervised machine learning to improve prognostic predictions for cervical cancer patients undergoing CCRT. By integrating radiomic features with inflammatory markers, this approach offered a more nuanced and precise method for patient stratification, which in turn could guide personalized treatment strategies. The promising results suggested that this technique could be crucial in enhancing patient outcomes, paving the way for more tailored and effective interventions in cervical cancer care.

## Data Availability

The raw data supporting the conclusions of this article will be made available by the authors, without undue reservation.

## References

[1] Sung H Ferlay J Siegel RL Laversanne M Soerjomataram I Jemal A. Global cancer statistics 2020: GLOBOCAN estimates of incidence and mortality worldwide for 36 cancers in 185 countries. CA Cancer J Clin. (2021) 71:209. doi: 10.3322/caac.21660 33538338

[2] Singh D Vignat J Lorenzoni V Eslahi M Ginsburg O Lauby-Secretan B. Global estimates of incidence and mortality of cervical cancer in 2020: a baseline analysis of the WHO Global Cervical Cancer Elimination Initiative. Lancet Glob Health. (2023) 11:e197. doi: 10.1016/S2214-109X(22)00501-0 36528031 PMC9848409

[3] Shrivastava S Mahantshetty U Engineer R Chopra S Hawaldar R Hande V. Cisplatin chemoradiotherapy vs radiotherapy in FIGO stage IIIB squamous cell carcinoma of the uterine cervix: A randomized clinical trial. JAMA Oncol. (2018) 4:506. doi: 10.1001/jamaoncol.2017.5179 29423520 PMC5885185

[4] Peters WA 3rd Liu PY Barrett RJ 2nd Stock RJ Monk BJ Berek JS. Concurrent chemotherapy and pelvic radiation therapy compared with pelvic radiation therapy alone as adjuvant therapy after radical surgery in high-risk early-stage cancer of the cervix. J Clin Oncol. (2023) 41:4605. doi: 10.1200/jco.22.02769 37797409

[5] Kastritis E Bamias A Efstathiou E Gika D Bozas G Zorzou P. The outcome of advanced or recurrent non-squamous carcinoma of the uterine cervix after platinum-based combination chemotherapy. Gynecol Oncol. (2005) 99:376. doi: 10.1016/j.ygyno.2005.06.024 16051322

[6] Schwarz JK Wahab S Grigsby PW. Prospective phase I-II trial of helical tomotherapy with or without chemotherapy for postoperative cervical cancer patients. Int J Radiat Oncol Biol Phys. (2011) 81:1258. doi: 10.1016/j.ijrobp.2010.07.038 20932657

[7] Marnitz S Köhler C Burova E Wlodarczyk W Jahn U Grün A. Helical tomotherapy with simultaneous integrated boost after laparoscopic staging in patients with cervical cancer: analysis of feasibility and early toxicity. Int J Radiat Oncol Biol Phys. (2012) 82:e137. doi: 10.1016/j.ijrobp.2010.10.066 21600704

[8] Marth C Landoni F Mahner S McCormack M Gonzalez-Martin A Colombo N. Cervical cancer: ESMO Clinical Practice Guidelines for diagnosis, treatment and follow-up. Ann Oncol. (2017) 28:iv72. doi: 10.1093/annonc/mdx220 28881916

[9] Gandy N Arshad MA Park WE Rockall AG Barwick TD. FDG-PET imaging in cervical cancer. Semin Nucl Med. (2019) 49:461. doi: 10.1053/j.semnuclmed.2019.06.007 31630730

[10] Grigsby PW. The prognostic value of PET and PET/CT in cervical cancer. Cancer Imaging. (2008) 8:146. doi: 10.1102/1470-7330.2008.0022 18694852 PMC2515618

[11] Kidd EA Siegel BA Dehdashti F Grigsby PW. The standardized uptake value for F-18 fluorodeoxyglucose is a sensitive predictive biomarker for cervical cancer treatment response and survival. Cancer. (2007) 110:1738. doi: 10.1002/cncr.22974 17786947

[12] Yang S Zhang Z Shen L. Prognostic significance of C-reactive protein in patients with cervical cancer: a meta-analysis. Front Oncol. (2023) 13:1232409. doi: 10.3389/fonc.2023.1232409 37731642 PMC10507700

[13] Han X Liu S Yang G Hosseinifard H Imani S Yang L. Prognostic value of systemic hemato-immunological indices in uterine cervical cancer: A systemic review, meta-analysis, and meta-regression of observational studies. Gynecol Oncol. (2021) 160:351. doi: 10.1016/j.ygyno.2020.10.011 33092868

[14] Skipar K Hompland T Lund KV Løndalen A Malinen E Kristensen GB. Risk of recurrence after chemoradiotherapy identified by multimodal MRI and 18F-FDG-PET/CT in locally advanced cervical cancer. Radiother Oncol. (2022) 176:17. doi: 10.1016/j.radonc.2022.09.002 36113778

[15] Ferreira M Lovinfosse P Hermesse J Decuypere M Rousseau C Lucia F. (18)F]FDG PET radiomics to predict disease-free survival in cervical cancer: a multi-scanner/center study with external validation. Eur J Nucl Med Mol Imaging. (2021) 48:3432. doi: 10.1007/s00259-021-05303-5 33772334 PMC8440288

[16] Isaji Y Tsuyoshi H Tsujikawa T Orisaka M Okazawa H Yoshida Y. Prognostic value of (18)F-FDG PET in uterine cervical cancer patients with stage IIICr allocated by imaging. Sci Rep. (2023) 13:18864. doi: 10.1038/s41598-023-46261-2 37914892 PMC10620427

[17] Zhang X Zhao J Zhang Q Wang S Zhang J An J. MRI-based radiomics value for predicting the survival of patients with locally advanced cervical squamous cell cancer treated with concurrent chemoradiotherapy. Cancer Imaging. (2022) 22:35. doi: 10.1186/s40644-022-00474-2 35842679 PMC9287951

[18] Liu Z Wang S Dong D Wei J Fang C Zhou X. The applications of radiomics in precision diagnosis and treatment of oncology: opportunities and challenges. Theranostics. (2019) 9:1303. doi: 10.7150/thno.30309 30867832 PMC6401507

[19] Ding C LT Peng W Park H. (2006). Orthogonal nonnegative matrix t-factorizations for clustering, in: Proceedings of the 12th ACM SIGKDD international conference on Knowledge discovery and data mining: ACM, New York, NY, USA: Association for Computing Machinery (ACM). pp. 126–35.

[20] Lambin P Leijenaar RTH Deist TM Peerlings J de Jong EEC van Timmeren J. Radiomics: the bridge between medical imaging and personalized medicine. Nat Rev Clin Oncol. (2017) 14:749. doi: 10.1038/nrclinonc.2017.141 28975929

[21] Verma V Simone CB 2nd Krishnan S Lin SH Yang J Hahn SM. The rise of radiomics and implications for oncologic management. J Natl Cancer Inst. (2017) 109. doi: 10.1093/jnci/djx055 28423406

[22] Shur JD Doran SJ Kumar S Ap Dafydd D Downey K O’Connor JPB. Radiomics in oncology: A practical guide. Radiographics. (2021) 41:1717. doi: 10.1148/rg.2021210037 34597235 PMC8501897

[23] Demircioğlu A. Benchmarking feature selection methods in radiomics. Invest Radiol. (2022) 57:433. doi: 10.1097/rli.0000000000000855 35045555

[24] Mayerhoefer ME Materka A Langs G Häggström I Szczypiński P Gibbs P. Introduction to radiomics. J Nucl Med. (2020) 61:488. doi: 10.2967/jnumed.118.222893 32060219 PMC9374044

[25] Guiot J Vaidyanathan A Deprez L Zerka F Danthine D Frix AN. A review in radiomics: Making personalized medicine a reality via routine imaging. Med Res Rev. (2022) 42:426. doi: 10.1002/med.21846 34309893

[26] Yusufaly TI Zou J Nelson TJ Williamson CW Simon A Singhal M. Improved prognosis of treatment failure in cervical cancer with nontumor PET/CT radiomics. J Nucl Med. (2022) 63:1087. doi: 10.2967/jnumed.121.262618 34711618 PMC9258568

[27] Lucia F Bourbonne V Pleyers C Dupre PF Miranda O Visvikis D. Multicentric development and evaluation of (18)F-FDG PET/CT and MRI radiomics models to predict para-aortic lymph node involvement in locally advanced cervical cancer. Eur J Nucl Med Mol Imaging. (2023) 50:2514. doi: 10.1007/s00259-023-06180-w 36892667

[28] Fang M Kan Y Dong D Yu T Zhao N Jiang W. Multi-habitat based radiomics for the prediction of treatment response to concurrent chemotherapy and radiation therapy in locally advanced cervical cancer. Front Oncol. (2020) 10:563. doi: 10.3389/fonc.2020.00563 32432035 PMC7214615

[29] Xu C Liu W Zhao Q Zhang L Yin M Zhou J. CT-based radiomics nomogram for overall survival prediction in patients with cervical cancer treated with concurrent chemoradiotherapy. Front Oncol. (2023) 13:1287121. doi: 10.3389/fonc.2023.1287121 38162501 PMC10755472

[30] Scapicchio C Gabelloni M Barucci A Cioni D Saba L Neri E. A deep look into radiomics. Radiol Med. (2021) 126:1296. doi: 10.1007/s11547-021-01389-x 34213702 PMC8520512

[31] Zhao M Gao Z Gu X Yang X Wang S Fu J. Predictive significance of lymphocyte level and neutrophil-to-lymphocyte ratio values during radiotherapy in cervical cancer treatment. Cancer Med. (2023) 12:15820. doi: 10.1002/cam4.6221 37325889 PMC10469726

[32] Ethier JL Desautels DN Templeton AJ Oza A Amir E Lheureux S. Is the neutrophil-to-lymphocyte ratio prognostic of survival outcomes in gynecologic cancers? A systematic review and meta-analysis. Gynecol Oncol. (2017) 145:584. doi: 10.1016/j.ygyno.2017.02.026 28222899

[33] Cho O Chun M Chang SJ. Exponential slope from absolute lymphocyte counts during radio-chemotherapy can predict an aggressive course of cervical cancer. Cancers (Basel). (2022) 14. doi: 10.3390/cancers14205109 PMC9600990 36291893

