# Supplementary material for: Harnessing unsupervised machine learning with [18F]FDG PET/CT to develop a composite model for predicting overall survival in cervical cancer patients undergoing concurrent chemoradiotherapy
Source: Front Oncol. 2025 May 2;15:1486654. doi: 10.3389/fonc.2025.1486654 (PMC12081247; doi:10.3389/fonc.2025.1486654)
Supplement: Supplementary file 1 [file Table1.docx]

**Table S1 The list of extracted radiomic features**

| **Shape(14)** | **First order(18)** |
| --- | --- |
| Elongation (Elongation)  Flatness (Flatness)  Least axis (LeastAxisLength)  Major axis (MajorAxisLength)  M2DDC (Maximum2DDiameterColumn)  M2DDR (Maximum2DDiameterRow)  M2DDS (Maximum2DDiameterSlice)  M3DD (Maximum3DDiameter)  Mesh volume (MeshVolume)  Minor axis (MinorAxisLength)  Sphericity (Sphericity)  Surface Area (SurfaceArea)  SVR (SurfaceVolumeRatio)  Voxel volume (VoxelVolume) | 10Percentile (10Percentile)  90Percentile (90Percentile)  Energy (Energy)  Entropy (Entropy)  Interquartile range (InterquartileRange)  Kurtosis (Kurtosis)  Maximum (Maximum)  MAD (MeanAbsoluteDeviation)  Mean (Mean)  Median (Median)  Minimum (Minimum)  Range (Range)  RMAD (RobustMeanAbsoluteDeviation)  RMS (RootMeanSquared)  Skewness (Skewness)  Total energy (TotalEnergy)  Uniformity (Uniformity)  Variance (Variance) |
| **GLCM(24)** | **GLDM(14)** |
| Autocorrelation (Autocorrelation)  Cluster prominence (ClusterProminence)  Cluster shade (ClusterShade)  Cluster tendency (ClusterTendency)  Contrast (Contrast)  Correlation (Correlation)  Difference average (DifferenceAverage)  Difference entropy (DifferenceEntropy)  Difference variance (DifferenceVariance)  Id (Id)  Idm (Idm)  Idmn (Idmn)  Idn (Idn)  Imc1 (Imc1)  Imc2 (Imc2)  Inverse variance (InverseVariance)  Joint average (JointAverage)  Joint energy (JointEnergy)  Joint entropy (JointEntropy)  MCC (MCC)  Max Probability (MaximumProbability)  Sum average (SumAverage)  Sum entropy (SumEntropy)  Sum squares (SumSquares) | Dependence entropy (DependenceEntropy)  DNU (DependenceNonUniformity)  DNUN (DependenceNonUniformityNormalized)  Dependence variance (DependenceVariance)  GLNU (GrayLevelNonUniformity)  GLV (GrayLevelVariance)  HGLE (HighGrayLevelEmphasis)  LDE (LargeDependenceEmphasis)  LDHGLE (LargeDependenceHighGrayLevelEmphasis)  LDLGLE (LargeDependenceLowGrayLevelEmphasis)  LGLE (LowGrayLevelEmphasis)  SDE (SmallDependenceEmphasis)  SDHGLE (SmallDependenceHighGrayLevelEmphasis)  SDLGLE (SmallDependenceLowGrayLevelEmphasis) |
| **GLRLM(16)** | **GLSZM(16)** |
| GLNU (GrayLevelNonUniformity)  GLNUN (GrayLevelNonUniformityNormalized)  GLV (GrayLevelVariance)  HGLRE (HighGrayLevelRunEmphasis)  LRE (LongRunEmphasis)  LRHGLE (LongRunHighGrayLevelEmphasis)  LRLGLE (LongRunLowGrayLevelEmphasis)  LGLRE (LowGrayLevelRunEmphasis)  Run entropy (RunEntropy)  RLNU (RunLengthNonUniformity)  RLNUN (RunLengthNonUniformityNormalized)  Run percentage (RunPercentage)  Run Variance (RunVariance)  SRE (ShortRunEmphasis)  SRHGLE (ShortRunHighGrayLevelEmphasis)  SRLGLE (ShortRunLowGrayLevelEmphasis) | GLNU (GrayLevelNonUniformity)  GLNUN (GrayLevelNonUniformityNormalized)  GLV (GrayLevelVariance)  HGZE (HighGrayLevelZoneEmphasis)  LAE (LargeAreaEmphasis)  LAHGLE (LargeAreaHighGrayLevelEmphasis)  LALGLE (LargeAreaLowGrayLevelEmphasis)  LGLZE (LowGrayLevelZoneEmphasis)  SZNU (SizeZoneNonUniformity)  SZNUN (SizeZoneNonUniformityNormalized)  SAE (SmallAreaEmphasis)  SAHGLE (SmallAreaHighGrayLevelEmphasis)  SALGLE (SmallAreaLowGrayLevelEmphasis)  Zone entropy (ZoneEntropy)  Zone percentage (ZonePercentage)  Zone variance (ZoneVariance) |
| **NGTDM(5)** |  |
| Busyness (Busyness)  Coarseness (Coarseness)  Complexity (Complexity)  Contrast (Contrast)  Strength (Strength) |  |

Shape (Shape Features); First order (First Order Features); GLCM (Gray Level Cooccurrence Matrix); GLDM (Gray Level Dependence Matrix); GLRLM (Gray Level Run Length Matrix); GLSZM (Gray Level Size Zone Matrix); NGTDM (Neighborhood Gray Tone Difference Matrix).

**Table S2 The list of selected features**

| **PET features** |
| --- |
| Elongation (Elongation)  Flatness (Flatness)  Least axis (LeastAxisLength)  Sphericity (Sphericity)  10Percentile (10Percentile)  90Percentile (90Percentile)  Kurtosis (Kurtosis)  Minimum (Minimum)  Cluster shade (ClusterShade)  Id (Id)  DNUN (DependenceNonUniformityNormalized)  LDLGLE (LargeDependenceLowGrayLevelEmphasis)  GLCM_Contrast (GLCM_Contrast)  Skewness (Skewness)  Correlation (Correlation)  MCC (MCC)  wavelet-LLH_glcm_Cluster shade (wavelet-LLH_glcm_Cluster shade)  wavelet-LLH_firstorder_Kurtosis (wavelet-LLH_firstorder_Kurtosis)  wavelet-LHL_firstorder_Median (wavelet-LHL_firstorder_Median)  wavelet-LHL_firstorder_Skewness (wavelet-LHL_firstorder_Skewness)  wavelet-LHL_glcm_Cluster shade (wavelet-LHL_glcm_Cluster shade)  wavelet-LHL_gldm_Dependence variance (wavelet-LHL_gldm_Dependence variance)  wavelet-LHH_firstorder_Kurtosis (wavelet-LHH_firstorder_Kurtosis)  wavelet-LHH_firstorder_Mean (wavelet-LHH_firstorder_Mean)  wavelet-LHH_glcm_Cluster tendency (wavelet-LHH_glcm_Cluster tendency)  wavelet-LHH_glcm_Correlation (wavelet-LHH_glcm_Correlation)  wavelet-LHH_glcm_Imc2 (wavelet-LHH_glcm_Imc2)  wavelet-LHH_gldm_LGLE (wavelet-LHH_gldm_LowGrayLevelEmphasis)  wavelet-LHH_glrlm_GLV (wavelet-LHH_glrlm_GrayLevelVariance)  wavelet-HLL_firstorder_Kurtosis (wavelet-HLL_firstorder_Kurtosis)  wavelet-HLL_firstorder_Range (wavelet-HLL_firstorder_Range)  wavelet-HLL_glcm_Cluster shade (wavelet-HLL_glcm_Cluster shade)  wavelet-HLL_glcm_Correlation (wavelet-HLL_glcm_Correlation)  wavelet-HLL_glrlm_HGLRE (wavelet-HLL_glrlm_HighGrayLevelRunEmphasis)  wavelet-HLH_firstorder_Median (wavelet-HLH_firstorder_Median)  wavelet-HLH_firstorder_Skewness (wavelet-HLH_firstorder_Skewness)  wavelet-HLH_glcm_Correlation (wavelet-HLH_glcm_Correlation )  wavelet-HLH_gldm_SDE (wavelet-HLH_gldm_SmallDependenceEmphasis)  wavelet-HLH_gldm_LGLE (wavelet-HLH_gldm_ LowGrayLevelEmphasis)  wavelet-HLH_glrlm_RunVariance (wavelet-HLH_glrlm_RunVariance)  wavelet-HHL_firstorder_Mean (wavelet-HHL_firstorder_Mean )  wavelet-HHL_firstorder_Maximum (wavelet-HHL_firstorder_Maximum) |
| **CT features** |
| \| Elongation (Elongation) \| \| --- \| \| Least axis (LeastAxisLength) \| \| 10Percentile (10Percentile) \| \| Kurtosis (Kurtosis) \| \| wavelet-LHL_firstorder_Mean (wavelet-LHL_firstorder_Mean) \| \| wavelet-LHH_glcm_Difference average (wavelet-LHH_glcm_Difference average) \| \| wavelet-LHH_firstorder_Median (wavelet-LHH_firstorder_Median) \| \| wavelet-HLL_glcm_Idn (wavelet-HLL_glcm_Idn) \| \| wavelet-HLH_glcm_MCC (wavelet-HLH_glcm_MCC) \| \| wavelet-HHL_glcm_InverseVariance (wavelet-HHL_glcm_InverseVariance) \| \| wavelet-HHH_firstorder_Mean (wavelet-HHH_firstorder_Mean) \| \| wavelet-LLL_firstorder_90Percentile (wavelet-LLL_firstorder_90Percentile) \| |

LLL: Represents low grayscale values in all three directions (low, low, low); LLH: Represents low grayscale values in the first two directions and high grayscale values in the third direction; LHL: Indicates low grayscale values in the first and third directions, and high grayscale values in the second direction; LHH: Represents low grayscale values in the first direction and high grayscale values in the second and third directions; HLL: Represents high grayscale values in the first direction and low grayscale values in the second and third directions; HLH: Indicates high grayscale values in the first and third directions, and low grayscale values in the second direction; HHL: Represents high grayscale values in the first two directions and low grayscale values in the third direction; HHH: Represents high grayscale values in all three directions. This indicates areas with very high contrast; firstorder (first order features); glcm (gray level cooccurrence matrix); gldm (gray level dependence matrix); glrlm (gray level run length matrix).
